# Simultaneous MR imaging for tissue engineering in a rat model of stroke

**DOI:** 10.1038/srep14597

**Published:** 2015-09-30

**Authors:** Francesca J. Nicholls, Wen Ling, Giuseppe Ferrauto, Silvio Aime, Michel Modo

**Affiliations:** 1 Department of Radiology, Pittsburgh, PA; 2 McGowan Institute for Regenerative Medicine and Pittsburgh, PA; 3 Department of Bioengineering, University of Pittsburgh, Pittsburgh, PA; 4 Institute of Psychiatry, Psychology and Neuroscience, King’s College London, UK; 5 Molecular Imaging Center, Department of Chemistry, University of Torino, Italy

## Abstract

*In situ* tissue engineering within a stroke cavity is gradually emerging as a novel therapeutic paradigm. Considering the varied lesion topology within each subject, the placement and distribution of cells within the lesion cavity is challenging. The use of multiple cell types to reconstruct damaged tissue illustrates the complexity of the process, but also highlights the challenges to provide a non-invasive assessment. The distribution of implanted cells within the lesion cavity and crucially the contribution of neural stem cells and endothelial cells to morphogenesis could be visualized simultaneously using two paramagnetic chemical exchange saturation transfer (paraCEST) agents. The development of sophisticated imaging methods is essential to guide delivery of the building blocks for *in situ* tissue engineering, but will also be essential to understand the dynamics of cellular interactions leading to the formation of de novo tissue.

Advances in regenerative medicine are increasingly providing new opportunities to repair damaged tissue. Nevertheless, a major challenge remains in replacing lost brain tissue, rather than merely implanting cells into existing tissue. An increased understanding of tissue formation during development, regeneration^1^ and *in vitro* organoids^2^,^3^ define necessary elements to achieve *in situ* tissue engineering^4^. The formation of de novo tissue, is dependent on the re-formulation of a neurovascular environment that is comprised of a neural and an endothelial element^5^,^6^,^7^. Although there is evidence that co-transplanted endothelial cells (ECs) improve the survival of neural stem cells (NSCs) and enhance their differentiation upon intraparenchymal implantation^8^, little is known about a reconstitution of a neurovascular environment within the lesion cavity.

Implantation of NSCs with a scaffold secreting vascular endothelial growth factor (VEGF) that attracted host endothelial cells to the cavity achieved a reconstitution of a neurovascular environment, but the pattern of vasculogenesis within the de novo tissue was heterogenous^9^. A faster and more homogenous vascularization of this de novo tissue could potentially be achieved by co-transplanting ECs and NSCs. The distribution of transplanted cells within the cavity and their interaction with the host brain are potentially pivotal to ensure the creation of a functional neurovascular tissue. Implanted cells can be pre-labeled with, for instance, ^19^F nanoemulsions to non-invasively monitor their distribution within this environment using MRI^10^. Indeed, differences in distribution are evident, ranging from areas lacking an interface with the host brain to cellular clumps, in addition to homogenous regions. To monitor *in situ* tissue engineering that is dependent on the interaction of multiple cell types, novel approaches are required to visualize the distribution of select populations of cells^11^,^12^,^13^.

Visualization of implanted cells has mainly relied on T2 agents that can achieve a high contrast and afford the tracking of a small population of cells^11^,^12^. As T1 and T2 agents within the same voxel cannot easily be separated, it is unlikely that this approach is amenable to report simultaneously on different cell populations. Although ^19^F-MRI can report simultaneously on two populations of cells within the same voxel^14^, its inherent sensitivity is low and it requires multi-nuclear MRI capabilities, hence limiting its wider applicability. In contrast, paramagnetic chemical exchange saturation transfer (paraCEST) affords a selective visualization of independent paraCEST agents with better sensitivity^15^,^16^. Indeed, paraCEST agents, Eu- and Yb-HPDO3A specifically, have been used to pre-label macrophages and tumor cells for proof-of-principle simultaneous *in vivo* visualization using MRI^17^,^18^. We here report the use of Eu- and Yb-HPDO3A to visualize simultaneously and non-invasively neural stem cells and endothelial cells after implantation into the stroke cavity.

## Results

### paraCEST agent detection

**Figure 1:**
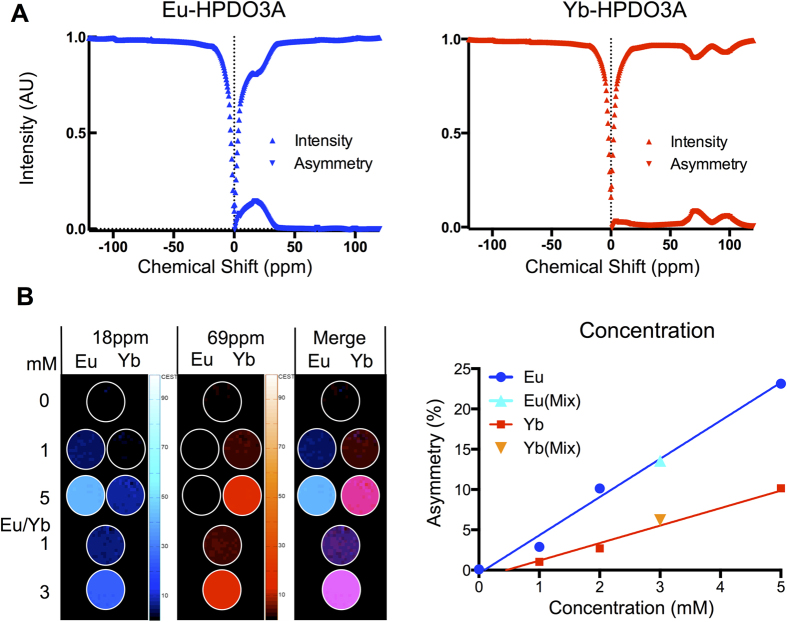
paraCEST agent detection. (**A**) z spectra (0.5 ppm intervals) of agent solutions (4 mM) showing signal intensity compared to the reference at −120 ppm for both Eu-HPDO3A (Eu) and Yb-HPDO3A (Yb). Asymmetry (subtraction of the intensity at each chemical shift from its corresponding negative) highlights the regions of chemical shift that can be exploited to achieve specific imaging of each agent. Eu and Yb regions are non-overlapping, hence affording selective imaging. (**B**) To determine if these agents afford selective, yet simultaneous imaging (i.e. occupying the same space), different concentrations of Eu and Yb were imaged at 18 ppm and 69 ppm respectively. Both agents provide a specific signal at either frequency. However, a concentration of 5 mM of Yb also produced a weak non-specific signal at 18 ppm. Although both agents induced a concentration-dependent linear change in asymmetry, the degree of asymmetry for Yb was significantly less than for Eu, a phenomenon that was accentuated at higher concentrations.

Simultaneous imaging of two populations of cells requires independent signals that can be separated and distinguished in the same space. Eu- and Yb-HPDO3A exhibit distinct chemical shifts that are evident on the MR signal after excitation at their specific frequency (Fig. 1A). In a 4 mM concentration solution (37 °C, pH 7.0), the resulting asymmetry indicates a single peak for Eu-HPDO3A at 18 ppm with an effect size of 15%, whereas Yb-HPDO3A possesses two exchange sites at 69 and 97 ppm. The 69 ppm site has the higher signal change with 8% effect size compared to 6% at 97 ppm. These properties can hence be exploited to selectively image Eu-HPDO3A at 18 ppm and Yb-HPDO3A at 69 ppm. Selectivity of these sites for imaging is evidenced by Eu- (Signal Intensity = −0.4127 + 4.739x[Eu]) and Yb-HPDO3A (Signal Intensity = −0.9877 + 2.171x[Yb]) only providing a dose-dependent signal at the relevant shift with a Eu/Yb mix being detectable at both (Fig. 1B). Indeed, irrespective of the agents being detected within a pure or a mixed solution, the extent of asymmetry increases in a linear fashion with Yb-HPDO3A being the less efficient agent. A higher concentration of Yb-HPDO3A is hence required to achieve the same level of asymmetry as Eu-HPDO3A. However, high concentrations (>5 mM) of Yb-HPDO3A also led to a negligible unspecific signal being detected at 18 ppm (Fig. 1B). Based on these results, an agent concentration of 1.14 mM for Eu-HPDO3A and 2.75 mM for Yb-HPDO3A per voxel is required to achieve a 5% signal change.

### Labelling of neural stem cells and endothelial cells

**Figure 2:**
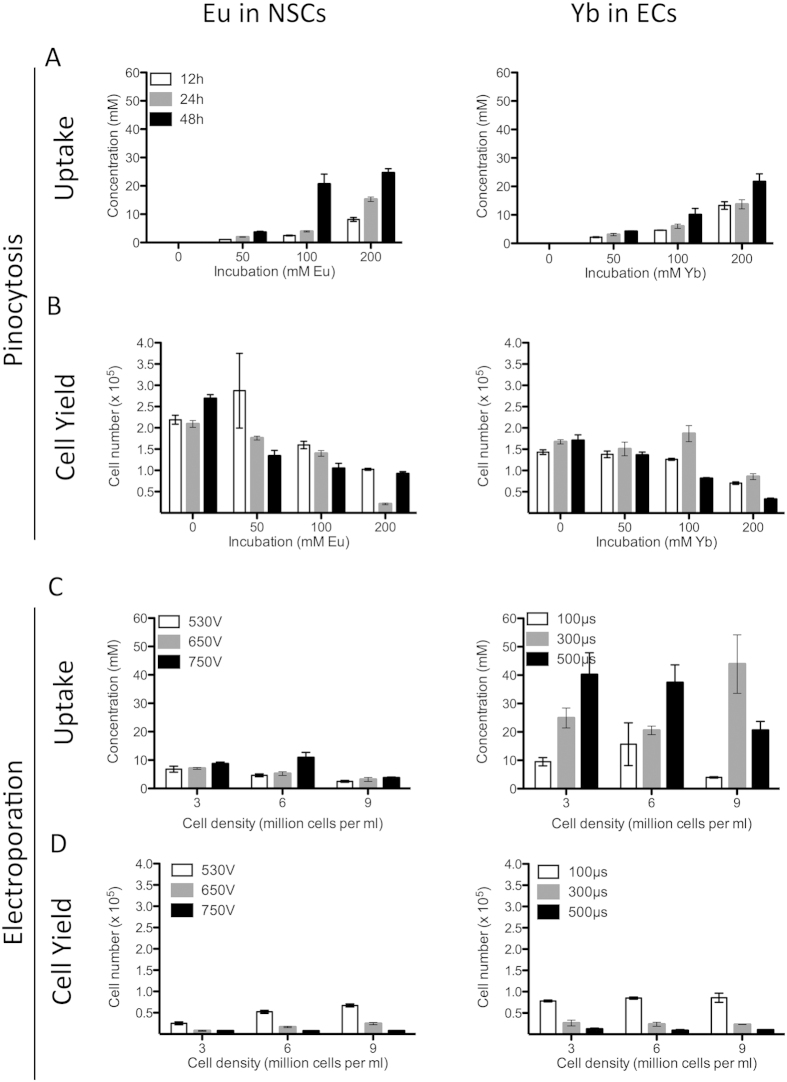
Cell labeling. (**A**) With pinocytosis, uptake was increased in both cell types by increasing concentration (F = 132, p < 0.0001 for NSCs and F = 101, p < 0.0001 for ECs) and incubation time (F = 72, p < 0.0001 for NSCs and F = 12, p < 0.001 for ECs), with a significant interaction between time and concentration (F = 20, p < 0.0001 for NSCs and F = 13, p < 0.05 for ECs). (**B**) Survival was decreased in both cell types by increasing concentration (F = 29, p < 0.0001 for NSCs and F = 64, p < 0.0001 for ECs) and incubation time (F = 6, p < 0.01 for NSCs and F = 22, p < 0.0001 for ECs), with a significant interaction between time and concentration (F = 4, p < 0.01 for NSCs and F = 8, p < 0.001 for ECs). (**C**) For electroporation in NSCs, uptake was increased by increasing voltage (F = 12, p < 0.001) and decreased by increasing cell density (F = 24, p < 0.0001). For ECs, uptake by electroporation was increased by increasing pulse length (F = 13, p < 0.0001), but was not affected by cell density (F = 0.1, p = 0.90). (**D**) In NSCs, survival after electroporation was decreased by increasing voltage (F = 259, p < 0.0001) but increased by increasing cell density (F = 55, p < 0.0001). For ECs, survival after electroporation was decreased by increasing pulse length (F = 197, p < 0.0001), but was not affected by cell density (F = 0.02, p = 0.98).

To achieve an intracellular agent concentration that affords detection, a significant uptake of agent is required for each cell type. However, as Eu-HPDO3A is a more efficient imaging agent, a lower intracellular concentration or fewer cells are required to warrant detection. Since NSCs are generally more susceptible to toxicity and less efficient in cellular uptake than ECs, NSCs were labeled with Eu-HPDO3A and ECs with Yb-HPDO3A. For pinocytosis, both NSCs and ECs showed increasing intracellular concentration of paraCEST agents with increasing incubation time and concentration (Fig. 2A), whereas cell yield (i.e. those cells that attached and survived for 24hrs) showed the opposite response, decreasing with increasing incubation time and concentration (Fig. 2B). Incubation times of at least 24 hours at 100 mM paraCEST agent concentration yielded cell uptake that was sufficient for imaging experiments, as measured by ICP-MS. Cell uptake of Eu was verifiable based on the fluorescent properties of this molecule (Supplementary Fig. 1A). However, a concern of pinocytosis is that agents are sequestered in endosomes that can affect its effect on water and hence affect chemical exchange leading to a quenching of the detectable signal.

In addition to pinocytosis, intracellular agent incorporation was also achieved using electroporation in an effort to ensure Eu- and Yb-HPDO3A being available in the cytoplasm at the highest possible concentration. Indeed, an efficient intracellular uptake was evident based on the fluorescence of Eu-HPDO3A (Supplementary Fig. 1B). ICP-MS indicated, however, that for NSCs pinocytosis uptake (up to 20 mM) is higher than electroporation (up to 10 mM), while preserving a better cell yield (Fig. 2C). In contrast, in ECs a significantly more efficient uptake of paraCEST agent was evident with 40 mM intracellular concentration. Still, electroporation impacted cell yield (87%) whereas no difference was seen with pinocytosis for conditions resulting in similar intracellular agent concentrations. Concept maps reveal that cell yield for NSCs (Suppl Fig. 1C) is the product of an interaction between cell density and electroporation pulse with intracellular concentrations being mostly affected by cellular density (Suppl Fig. 1D). Electroporation conditions of 530 V with a 100 μs pulse at an NSC density of 3 × 10^6^ cells/mL resulted in a 6.8 mM concentration. In contrast, for ECs, pulse length mostly determines cell yield (Suppl Fig. 1E) and intracellular concentration of Yb-HPDO3A (Suppl Fig. 1F). Using the same conditions as for NSCs, Yb-HPDO3A results in a 9.5 mM intracellular concentration for ECs. Electroporation did not significantly affect proliferation in NSCs (Suppl. Fig. 1G), but at 7 days led to a significant reduction in proliferation of ECs (p < .001, Suppl. Fig. 1H). These conditions hence provide fairly well matched conditions for both cell types.

### Establishing cellular detection using paraCEST

**Figure 3:**
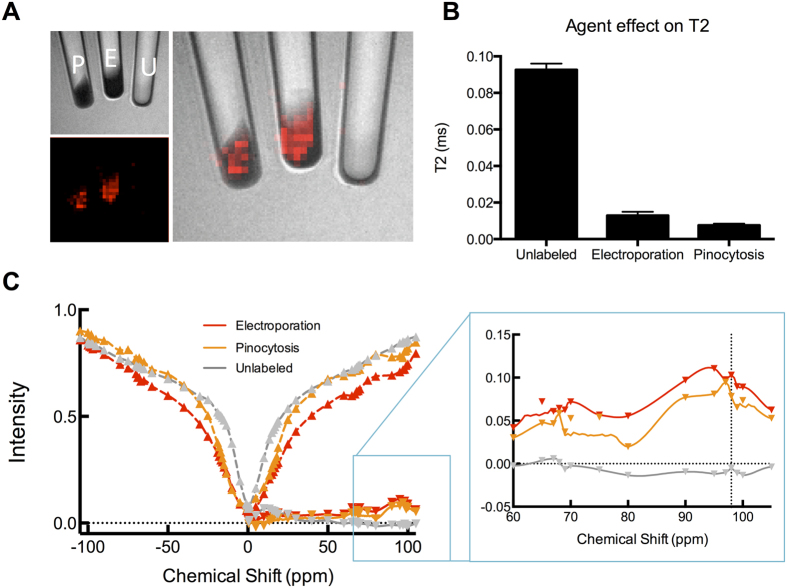
MRI detection of cells labeled via pinocytosis or electroporation. (**A**) Cells labeled with Yb-HPDO3A via electroporation (530 V, 100 μs pulse to give 9.5 mM pellet) (E) and pinocytosis (100 mM, 24 h to give 10.1 mM pellet) (P) were readily detected whereas unlabeled cells (U) did not produce a significant asymmetry. However, the asymmetry effect of Yb-HPDO3A is markedly attenuated after cell uptake compared to its effect in solution. (**B**) It was also evident that Yb-HPDO3A induced a dramatic shortening of T2 with a more significant shortening being evident upon pinocytotic incorporation. (**C**) Electroporation of Yb-HPDO3A showed a more significant asymmetry at 97 ppm than those labeled via pinocytosis. Notably, the asymmetry at 69 ppm was very much reduced. Z spectra were generated using image acquisitions at 66–71 and 95–100 ppm at 1 ppm per step, with reference images at 0 and −300 ppm. The remaining data points were fitted using smoothing splines.

Since paraCEST agents are highly pH sensitive, intracellular localization to the endosomes (as in the case of pinocytosis) and subsequent fusion with low pH lysosomes is likely to reduce detection. To assess this, ECs were labeled with Yb via pinocytosis (10.1 mM) and electroporation (9.5 mM). Both methods of incorporation produced cell-specific detectable signal changes on the CEST and T2 scan (Fig. 3A). A strong T2 shortening effect was evident for all labeled cells and this was more pronounced for those labeled through pinocytosis (Fig. 3B). Nevertheless, this T2 effect does not interfere with paraCEST imaging, provided a very short TE is used (<10 ms). Within cells, the 97 ppm exchange site revealed a greater asymmetry (10%) than 69 ppm (6%) (Fig. 3C). Despite the pinocytosis pellets having a 5% higher intracellular concentration, electroporated cells generated a 2% stronger signal at 97 ppm. Changes in microenvironment due to differential uptake therefore affect the paraCEST signal, but not in a dramatic fashion. Nevertheless, incorporation of agent into cells reduced signal for ~10 mM Yb concentration from 21% in solution to 6% in cells hinting at other factors that attenuate detection.

### Magnetization Transfer (MT) Effects

**Figure 4:**
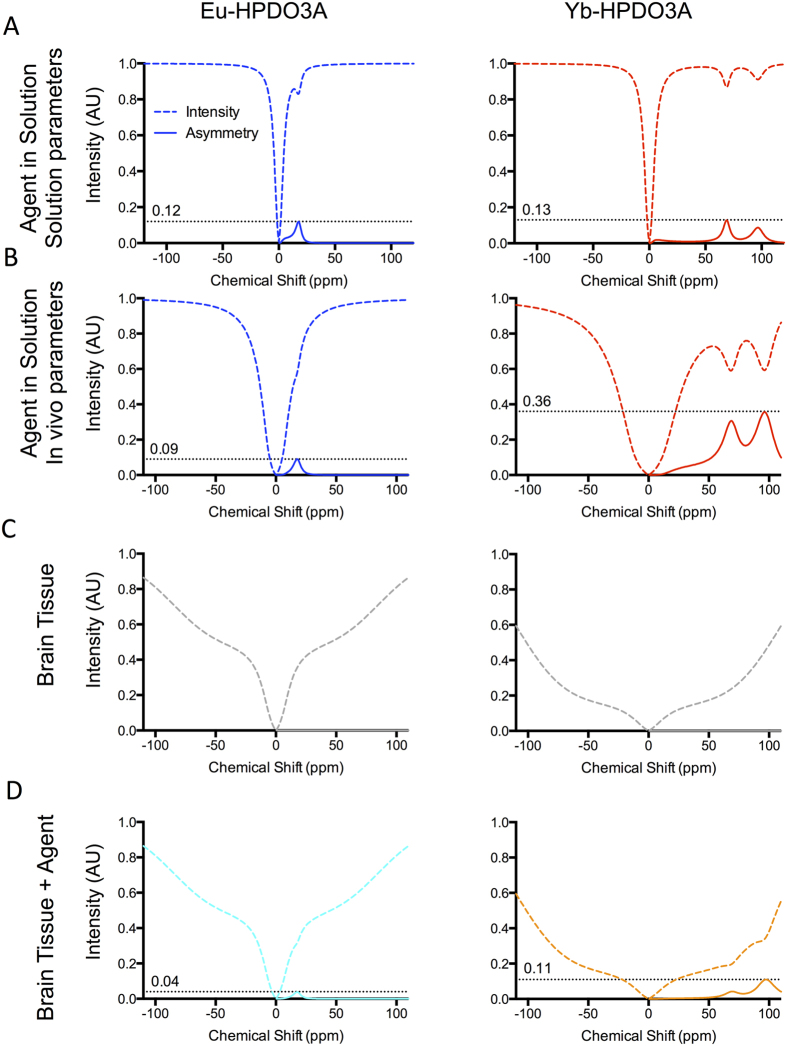
Magnetization Transfer (MT) effect simulation. (**A**) Numerical calculation of Bloch-McConnell equation shows similar z spectra to experimentally acquired data of agent in solution (1.5 s, 15 μT presaturation pulse for Eu, and 800 ms, 23 μT pulse for Yb). (**B**) When the pulses are altered (to 800 ms, 28 μT for Eu and 600 ms, 56 μT for Yb) to provide improved contrast *in vivo*, the water peak becomes wider, but contrast achieved for Yb is significantly increased. (**C**) MT effects in brain tissue further widen the water peak, (**D**) resulting in decreased agent contrast.

Spectra acquired in solution typically have a narrow water peak at 0 ppm with clearly defined peaks associated with each agent, whereas in cells the water peak becomes much wider due to MT effects. To investigate these effects and optimize detection, a Bloch simulation modeling these effects was calculated using parameters estimated based on z spectra measured for Eu-HPDO3A and Yb-HPDO3A in solution (Suppl Table 1). Solution spectra reflect the asymmetries observed experimentally for concentrations equivalent to those in cell pellets (Fig. 4A). These notably demonstrate a narrow water peak with specific exchange sites. When RF conditions are altered to those used *in vivo*, the water peak marginally widens due to the increased power, but a significant increase in agent contrast is visible for Yb (Fig. 4B). The same RF conditions on brain tissue reveals a much broader water peak (Fig. 4C). As Yb-HPDO3A requires a higher RF power, the water peak is broader than for Eu-HPDO3A, even in the absence of any paraCEST agent. Once the paraCEST agents are added to the simulations, the widened water peak due to brain tissue causes additional significant loss of signal (Fig. 4D). Specifically, the simulation revealed that MT effects account for the signal loss seen experimentally in Eu-HPDO3A from 31% to 1.1% at 6.8 mM concentration and from 21% to 10% for a 9.5 mM of intracellular Yb-HPDO3A. This shows that MT effects have a much more significant impact on Eu than Yb, due to the closer proximity to the water peak. This makes Yb more sensitive in cells, whereas Eu is more sensitive in solution. A simultaneous *in vivo* detection therefore will depend on the detection of low levels of asymmetry of high concentrations of paraCEST agent.

### Simultaneous detection of NSCs and ECs

For the simultaneous detection of two populations of cells within the same voxel, a sufficient concentration of paraCEST agent inside each population needs to be achieved. To achieve a neurovascular reconstitution, a ratio of 4:1 NSCs:ECs is appropriate. This ratio will result in 4.5 mM of Eu-HPDO3A and 1.6 mM of Yb-HPDO3A within a voxel, if there is a homogenous mixing of cells and PBS (160,000 NSCs versus 40,000 ECs) within a given space. It is important to note that for instance for ECs, this reduces the number of ECs per voxel by 80% compared to only ECs being contained within a voxel.

**Figure 5:**
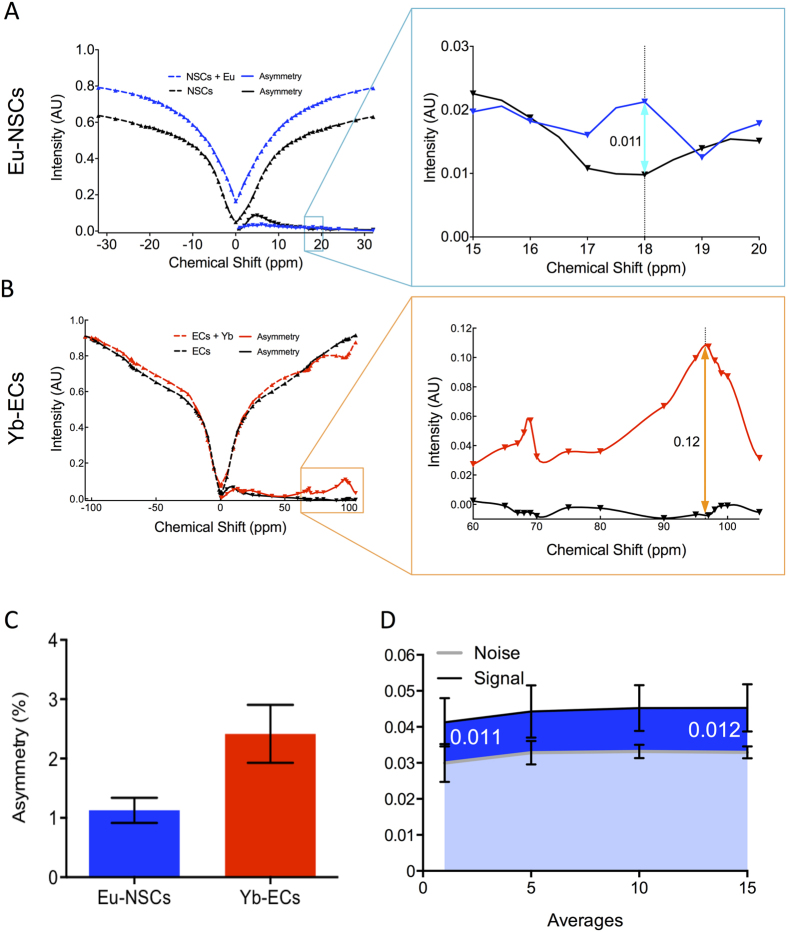
*In vitro* imaging of cell mixtures. (**A**) To determine if the required cell ratio will afford visualization, Eu-HPDO3A-labeled and unlabeled NSCs pellets were scanned *in vitro* to reveal a 1.1% difference in signal. (**B**) A 12% signal difference was evident between Yb-HPDO3A-labeled and unlabeled ECs. (**C**) At a cell mixture of 1 EC for every 4 NSCs, a signal asymmetry of 1.1% was maintained for Eu-labeled NSCs, but due to the lower abundance of Yb-HPDO3A-labelled ECs only a 2.3% effect was detected. (**D**) A 1.1% signal for Eu-HPDO3A-labelled cells was consistent, but a reliable distinction from noise required >5 signal averages.

In cell pellets of a single cell type, NSCs labeled with Eu-HPDO3A produce a signal differential of 1.1% compared to unlabeled NSCs (Fig. 5A). In contrast, the differential between Yb-HPDO3A and unlabeled ECs was 12% (Fig. 5B). A homogenous 4:1 NSC mixture resulted in contrast of 1.1% for Eu-HPDO3A and 2.4% for Yb-HPDO3A labeled cells compared to an unlabeled control cell mixture (Fig. 5C). As the effect size of the asymmetry is low, a key aspect is the variability of the signal to afford a reliable distinction between labeled and unlabeled cells. Increasing the number of acquisition averages to 10, reduced variability while maintaining the same effect size (Fig. 5D). Nevertheless, a significant difference between paraCEST labeled and unlabeled cells can be achieved within the same voxel.

### *In vivo* distribution of implantation NSCs and ECs

**Figure 6:**
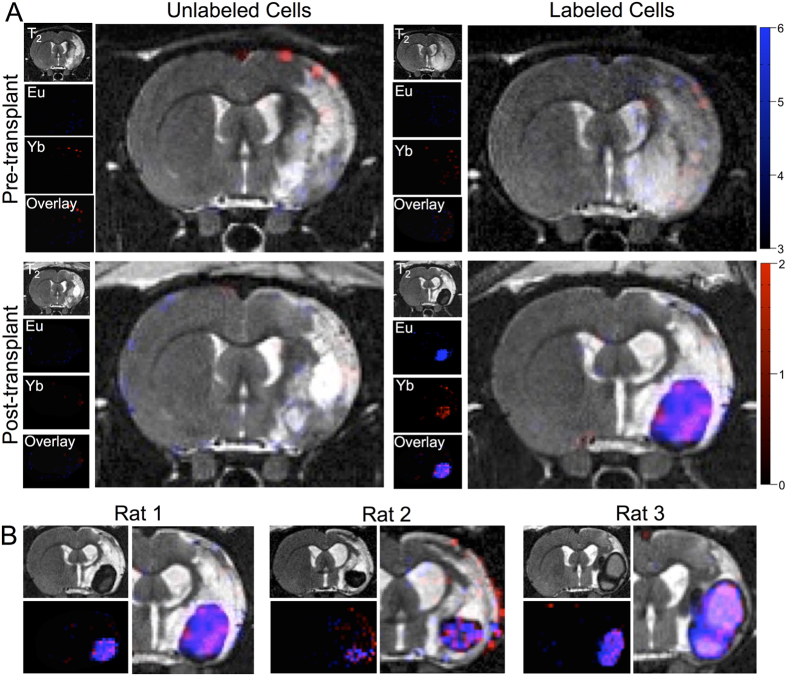
*In vivo* imaging of the distribution of implanted NSCs and ECs. (**A**) Pre-transplant baseline images indicated a few individual voxels with weak asymmetries at both 18 ppm (blue) and 97 ppm (red). Upon transplantation of unlabeled NSCs (160,000 cells) and ECs (40,000 cells), a few sporadic voxels could be observed, at similar levels to baseline. In contrast, upon implantation of Eu-HPDO3A and Yb-HPDO3A labeled NSCs and ECs, a clear localized signal at both 18 ppm and 97 ppm was evident with fewer unspecific voxels (due to better shimming to the actual signal). A T2 signal decrease was also evident due to the lanthanide complexes. Color-coded scale bars indicate % asymmetry. (**B**) These results are representative of all animals in both groups. In animals with labeled cells this afforded a visualization of the relative distribution of NSCs and ECs within the lesion cavity. In one animal, further unspecific signal at 97 ppm was observed highlighting the need for further improvements in paraCEST acquisition as well as image processing.

To assess the potential of paraCEST to simultaneously visualize the distribution of NSCs and ECs *in vivo*, labeled and unlabeled cells were implanted into the stroke cavity of rats two weeks after middle cerebral artery occlusion (MCAO) surgery. Pre-transplant a few unspecific voxels were apparent (Fig. 6A). After transplantation of unlabeled cells, a few sporadic voxels exhibiting MT effects were evident. Implantation of paraCEST-labeled cells in contrast resulted in a dramatic decrease in T2 in the area of transplantation, but also produced a robust signal at 18 ppm (Eu-HPDO3A) and 97 ppm (Yb-HPDO3A) at the site of implantation. Although a homogenously mixed population of NSCs and ECs was implanted, regional differences in distribution were apparent after 24 hours survival. This pattern of *in vivo* representation of the distribution of NSCs and ECs was apparent in all 3 animals indicating the reproducibility of this approach (Fig. 6B). However, it is noteworthy that in one subject (rat 2), a significant number of unspecific voxels at 97 ppm were detected. It is important to note that a lack of signal in a region, does not necessarily imply the absence of one particular cell type, but is likely a reflection of certain regions having more cells than other regions. The average signal difference between labeled and unlabeled cells in the lesion cavity was 3.27% ± 0.78 (p < 0.05) for Eu-HPDO3A and 1.16% ± 0.19 (p < 0.01) for Yb-HPDO3A. From the overlay images, it is also apparent that implantation of an NSC/EC mix did not sufficiently disperse to cover the stroke-damaged area.

**Figure 7:**
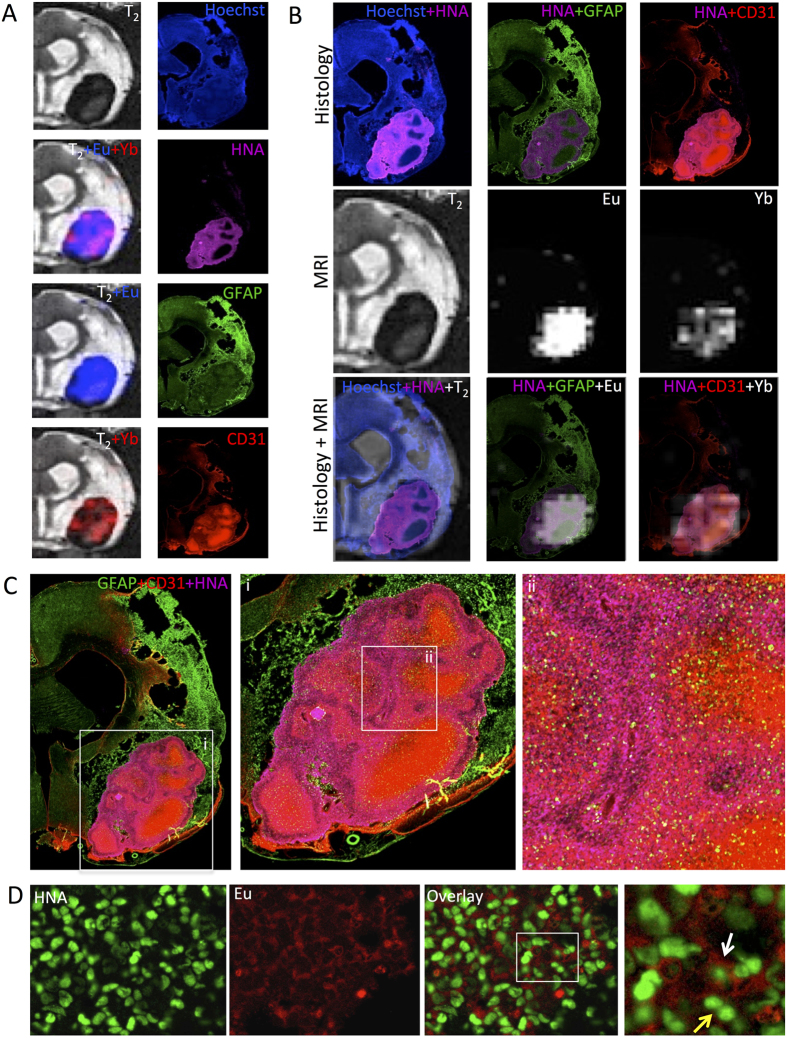
Histological validation of *in vivo* imaging. (**A**) There is a good correspondence between the distribution of transplanted cells as visualized by paraCEST and the histological marker for human cells (Human Nuclei Antigen, HNA). Indeed, the Eu (18 ppm) image indicated a fairly homogenous distribution of NSCs, which was paralleled by its histological validation using GFAP as a marker within the transplant area. Yb (97 ppm) imaging also truthfully reflects the macroscopic distribution of transplanted ECs, as detected by CD31. (**B**) To further validate the accuracy of the MR images, partially transparent histological overlays of transplanted cells (HNA) with NSCs (GFAP) and ECs (CD31) specific markers were co-registered based on landmark identification to the relevant MRI images. The images further highlight the regional specificity of the MR images as well as a correct mapping of relative cell distribution. (**C**) Further histological analyses indicated a morphological formation within the grafted area that reflects the emergence of a de novo neurovascular environment resembling structures observed in organoids. (**D**) Within this environment, the presence of Eu-HPDO3A in transplanted NSCs was also validated using its fluorescent properties.

A histological validation of *in vivo* MR images indicates that *in vivo* images truthfully represent the presence and distribution of transplanted human cells (Fig. 7A). Indeed, the T2 signal decrease due to the incorporation of the paraCEST agents resulted in an overall demarcation of implanted cells that corresponded to the distribution of human cells within the rat brain (Fig. 7B). Eu-HPDO3A consistently represented the distribution and regional patterning of implanted neural stem cells, whereas Yb-HPDO3A reflected the pattern of ECs. Histology and MRI overlays further demonstrate the dependability of *in vivo* images to report on the histological reality. Implanted cells were contained within the lesion cavity that is embedded within damaged tissue. However, as indicated on the paraCEST scans, human cells did not invade this tissue. A tissue patterning revealed on paraCEST scans as differences in regional distributions of NSCs and ECs resembled the generation of major vascular structures with neural patches, but there was no direct evidence of neurovascular environment forming yet. The cell density within the implant region exceeded that of surrounding healthy tissue, potentially reflecting that a too dense mixture of cells is being injected or that insufficient dispersion of cells into surrounding damaged tissue occurred (as the whole T2 hyperintense area was used to calculate the volume for injection). Finally, the presence of Eu-HPDO3A, as source of the paraCEST signal, was validated in human cells (Fig. 7D). These results demonstrate the feasibility to simultaneously image non-invasively two populations of cells that putatively occupy the same space in an *in situ* tissue engineering paradigm, but they also highlight the technical challenges that need to be addressed to make this approach more amenable to answer key biological questions.

## Discussion

The emergence of *in situ* tissue engineering to repair damaged brain tissue requires the development of novel non-invasive imaging techniques that can selectively visualize different cellular fractions^11^. The imaging requirements for tissue engineering are significantly different from monitoring the migration of cells within tissue. Specifically, paramagnetic CEST (paraCEST) contrast agents present a unique opportunity to visualize simultaneously multiple populations of cells and to report on their distribution within soft tissue^17^. Indeed, this approach here allowed us to image *in vivo* the distribution of human neural stem cells, as well as brain endothelial cells, that were co-implanted into a stroke cavity to form de novo brain tissue.

A key aspect of *in situ* tissue engineering is to create a vascularized tissue^9^. In the brain, the basic physiological entity to be replaced is the neurovascular unit^19^, which depends on endothelial cells forming a vascular network and neural stem cells developing the neuropil^7^. Indeed, we here observed a morphogenesis that resulted in a relative shift of distributions of cell populations after injection leading to neural patches and elongated structures of cell phenotypes within the lesion cavity. We previously reported the same phenomenon using these two types of cells *in vitro*, where the combination of these two cell populations lead to the creation of a neurovascular environment akin to those observed in brain tissue^5^. A basic neurovascular environment hence was re-created here and its morphological organization could be visualized using paraCEST imaging. Still, it remains unclear if this de novo tissue can survive long-term; if indeed it will develop an integrated functional vasculature and if NSCs will indeed develop into a functional tissue. Longer-term experiments will be required to ascertain these biological aspects.

Although histology will be required to answer many of these biological questions in detail, an adequate delivery and distribution within the lesion environment is essential prior to investigation of these biological aspects. Certainly, we have demonstrated that paraCEST imaging allowed us here to visualize the presence of implanted cells within the lesion environment. Indeed, implanted cells here were confined to the cavity, although T_2_-weighted imaging revealed a more extensive damaged tissue, which the implanted cells did not invade. Furthermore, paraCEST imaging here allowed us to visualize the relative distribution of two populations of cells within the graft demonstrating that these underwent morphogenesis akin to what was observed *in vitro*, rather than being homogenously distributed within the graft. As evidenced here, each animal presented with a unique lesion topology that evolves over time and interacts with the implanted cells. Histological analyses are inadequate to monitor these aspects and their interaction over time. Non-invasive imaging will therefore be essential to address how injected cells gradually distribute within the lesion environment and how they interface with host tissue. Indeed, these two key questions can be answered using the methodology described here.

Still, paraCEST imaging encounters major challenges for a long-term reliable imaging. Foremost of all, sensitivity of detection is low. Although a significant amount of paraCEST agents can be incorporated into both cell types, the detection threshold is high and hence voxel size needs to be relatively large. This approach is hence only suitable for monitoring the distribution of cell populations, akin to the use of 19F agents^10^,^20^. A major factor in the low sensitivity is that intracellular incorporation dramatically reduces the effect of paraCEST agents by affecting MT asymmetry, which is thought to arise from a shift difference between the solid-like macromolecular chemical shift center and bulk water resonance^21^. These cause a reduction in contrast compared to the same concentration in solution. Although increasing the power of the saturation pulse can increase contrast, it also increases MT and SAR effects. Further issues that need to be addressed are that MT effects in tissue lead to a widening of the water peak, i.e. reducing the specificity of excitation, and interfering with a robust detection of paraCEST agent with resonances close to the water peak. Improving intracellular location and concentration, as well as optimizing the exchange rate properties^22^ of the agents are therefore attractive options to improve the detection and applicability of paraCEST agents.

However, Schmidt *et al.*^23^ recently described an alternative approach using Tm- and Dy-DOTMA agents that are highly shifted, hence avoiding MT effects close to the water peak. Furthermore, a direct detection of the agent was described, rather than measuring exchange effects, hence yielding greater specificity. Still, sensitivity remains at a similar population level, suitable for application in tissue engineering paradigms. This level of resolution is sufficient to monitor macroscopic distributions, but greater detail of how individual populations interact with each other, as well as damaged tissue, will eventually be required. Higher sensitivity might eventually be achieved by incorporating novel classes of agents, such as shape-changing nanoprobes^24^ or xenon hyper-CEST^25^ that can potentially create many different selectively detectable signals integrated into multi-color images of tissue-engineered environments. The development of multiple high contrast CEST reporter genes would allow us to ensure that only viable cells are imaged^26^. The design of tissue engineered constructs should hence not only consider a simple cell imaging, but also the incorporation of additional extracellular probes that could report on, for instance, tissue metabolism^27^ and viability^28^. The development of multiple highly sensitive CEST reporters could indeed provide new avenues to achieve these requirements^29^,^30^. Nevertheless, although these approaches generate excitement for future applications, none of these approaches has so far been demonstrated to visualize two populations of cells occupying the same space *in vivo*.

*In situ* tissue engineering therefore provides a novel challenge to non-invasive imaging, which significantly differs from those applied to cell tracking. Specifically, a large population of cells is required to replace lost tissue. Multiple populations of cells are required to engineer a new tissue and ideally each is visualized selectively, hence reducing the amount of signal per population of cells per voxel volume. Affording simultaneous visualization of cells within the same space requires contrast mechanisms that do not interfere with each other. *In situ* tissue engineering for brain repair offers new opportunities, but also poses new challenges for non-invasive imaging techniques. A concerted effort is hence vital to provide the appropriate imaging tools to enable this new prospective therapeutic approach.

## Methods

### paraCEST agent synthesis

The synthesis of Yb(III)- and Eu(III)-complexes was achieved by mixing either Yb_2_O_3_ or Eu_2_O_3_ (Sigma-Aldrich) with the ligand HPDO3A (1:2 molar ratio, kindly provided by Bracco Imaging) in water. The mixture was left to react for 2 weeks under stirring and heating at 80 °C. The purity of the compounds is ~90%, as evaluated by its bulk magnetic (^1^H) susceptibility (BMS) shifts in solution (tert-butanol in water) in comparison to the same solution without a paramagnetic compound (Evans’ Method)^31^.

### Cell culture

The human striatal neural stem cell (NSC) line STROC05 (kindly provided by ReNeuron, UK) and the human cerebral microvascular endothelial cell hCMEC/D3 (kindly provided by P-O Couraud, Institut Cochin, France) were cultured and passaged as previously described in detail^5^. In brief, STROC05 NSCs are conditionally immortalized under the control of 4-hydroxytamoxifen (100 nM; Sigma), in the absence of which they will cease proliferation and undergo differentiation^32^. NSCs were expanded on laminin (10 μg/mL) coated flasks. Recombinant human basic fibroblast growth factor (bFGF; 10 ng/mL; PeproTech) and epidermal growth factor (EGF; 20 ng/mL; PeproTech) were used as mitogens. NSCs were passaged and used for experiments when they reached 70–80% confluency. D3 microvascular endothelial cells (ECs) were expanded on rat tail collagen type I (150 μg/mL) coated flask in the presence of 5% fetal bovine serum (PAA)^33^. ECs were passaged at 95% confluency. All culturing was performed at 37 °C in 5% CO_2_.

### Selection of cell labeling parameters

#### Pinocytosis

Cells were incubated in their normal growth medium containing Eu-HPDO3A for NSCs or Yb-HPDO3A for ECs at 0, 50, 100 or 200 mM and incubated for 12, 24 or 48 hours before being washed 3x HBSS (Gibco) and harvested using Accutase (Sigma).

#### Electroporation

To determine optimal parameters, a range of electroporation conditions was evaluated for NSCs and ECs using carboxyfluorescein (Sigma) as a proxy agent in hypo-osmolar buffer in 2 mm cuvettes using a Multiporator (Eppendorf). It was evident that uptake into NSCs was highly influenced by voltage, whereas uptake into ECs was subject to pulse length (Supplementary Fig. 2). To achieve an efficient uptake of paraCEST agents into these cell types, these two parameters (voltage and pulse length) were adjusted. Notably, for Eu-HPDO3A uptake into NSCs, voltages of 530 V, 650 V and 750 V were applied with a pulse length of 100 μs. In contrast for Yb-HPDO3A uptake into ECs, a 530 V voltage was combined with pulse lengths of 100, 300 and 500 μs. As uptake into either cell type is also dependent on cell densities, these were arrayed at 3, 6 and 9 × 10^6^ cells/mL. Cells were plated overnight before being washed 3x HBSS and harvested using Accutase.

#### Assessment

Number of cells harvested was counted using a haemocytometer (4 counts per sample; n = 3 samples) as a measure of cell yield. Cells were then analysed for Lanthanide content using ICP-MS.

### Inductively Coupled Plasma Mass Spectrometry (ICP-MS)

The Lanthanide content of cells was determined using ICP-MS. Cells were collected in 0.1 mL PBS and destroyed by sonication before being digested with concentrated HNO_3_ (70%) under microwave heating (MicroSYNTH). After mineralization, each sample was taken with 2 mL of ultrapure water and analyzed using an ELEMENT 2 ICP-MS (Thermo Scientific). Three replicates of each sample solution were analyzed.

### Optimized cell labeling protocol

Cell labeling requires an uptake of agent that affords detection, but also a sufficient yield of viable cells is essential to ensure its potential for cellular MRI^12^. Here, electroporation ensured an efficient agent uptake that preserved its imaging characteristics, but also resulted in a sufficient number of viable cells for transplantation. Eu-HPDO3A or Yb-HPDO3A agent (200 mM) were electroporated (530 V, 100 μs pulse length) at a cell density of 3 × 10^6^ cells/mL. After electroporation, cells remained in the electroporation solution for 12 min at room temperature to allow agent diffusion and pore closure. The suspension was then removed from the cuvette and diluted in pre-warmed fresh growth media at 1:15 dilution. Cells were then plated and allowed to attach overnight before being washed and harvested for imaging or implant.

To determine if the optimized cell labeling procedure affected proliferation, NSCs and ECs were labeled and fixed with 4% paraformaldehyde at 1 or 7 days (in non-proliferative conditions). The control condition consisted of non-electroporated cells. Coverslips were rinsed 3x with PBS prior to overnight application of a rabbit anti-Ki67 (1:400, Abcam, ab15580). The following day, coverslips were rinsed with PBS and a secondary antibody Alexa488 (Molecular Probes) was applied for 60 min prior to 3 rinses with PBS and coverslipping with Vectashield for fluorescence containing DAPI. Cell counts were performed using CellProflier version 2.1.1^34^.

### paraCEST Imaging

#### Hardware

MR Images were acquired on a 9.4 T horizontal bore Varian system equipped with an Agilent VnmrJ 3.1 console. A custom made volumetric birdcage quadrature coil (Virtumed LLC) achieving a two channel (0° and 90°) radiofrequency (RF) power input of up to 55 μT/5 sec with an internal diameter of 36 mm and effective length of 25 mm designed to achieve a high B1 homogeneity was used for all imaging.

#### In vitro agent and cell phantom imaging

For agent only phantoms, agents were diluted to the required concentration in PBS and the resulting solution was placed into 0.1 mL PCR tubes (Axygen). These tubes were then placed into a cylinder of Agar gel (2%) for imaging. For cell pellet phantoms, cells were washed three times in HBSS, harvested using Accutase, re-suspended in DMEM/F12 for counting and then mixed at the appropriate ratio if applicable before spinning down and re-suspending in a small volume of PBS for transfer into 0.1 mL PCR tubes. These were then spun down to produce a cell pellet, and tubes were placed into Agar as for solutions. *In vitro* imaging was carried out at room temperature. T_2_w images were acquired using Fast Spin Echo Multi Slice (FSEMS: TR = 6.1 s, TE = 70.78 ms, n = 8, 30 × 30 mm FOV, 192 × 192 matrix, 0.5 mm slice thickness).

#### z spectra and exchange rates

To define initial acquisition parameters for solution experiments, arrays of power, repetition time and pulse length were conducted to determine their effect on signal-to-noise ration (SNR), contrast-to-noise (CNR) which was defined as the percentage of CEST asymmetry, as well as the specific absorption rate (SAR) to define potential effects on sample temperature (Supplementary Fig. 3M). Based on contour maps of these arrays and measurements, a Fast Spin Echo (FSE) sequence (30 × 30 mm FOV, 64 × 64 matrix, 1 mm slice thickness, single slice) for Eu-HPDO3A (TR = 3 s, TE = 4.2 ms) and Yb-HPDO3A (TR = 8 s, TE = 8.4 ms) was preceded by a saturation scheme consisting of a continuous rectangular wave pulse. To calculate z spectra, frequency offset ranges of ±110 ppm were acquired at 5 ppm intervals. An additional reference offset at ±300 ppm was acquired for curve fitting. This acquisition paradigm did not affect cell viability up to 5 hours of continuous scanning (Supplementary Fig. 4A).

#### MT simulation

It has been reported that the applicability of the paraCEST technique for cell labeling in brain can be seriously undermined by the existence of broad MT (Magnetization Transfer) effects (from −100 ppm to 100 ppm)^35^. When in solution, paraCEST agents interact with bulk free water (T2 of ~40 ms), but upon incorporation into cells they interact to a lesser effect with bulk free water and more with water molecules bound in proteins (T2 of ~ 9.2 μs). In the presence of cells and tissue, the additional pool of bound water molecules hence also exchanges with the free bulk water. The additive effect is that cells and tissue will produce a MT effect. Therefore, even in the absence of paraCEST agent, CEST effects occur in tissue.

The choice of labeling sites cannot be fully understood without full z-spectra. Although these were acquired for paraCEST agents in solution, they are impractical to generate experimentally within a physiologically-feasible *in vivo* scanning time. It is important to note that a full characterization of the exchange properties of these agents is beyond the scope of this report, as it would also require an investigation of the effects of the chelate^36^, but reasonable approximations can be made that allow a simulation of the dominant MT effects that contrast CEST effects of paraCEST agents in solution versus *in vivo*. An MT influence on paraCEST agents can, nevertheless, be estimated by a Bloch simulation. 2− (Eu-HPDO3A) and 3-pool (Yb-HPDO3A) Bloch simulations were conducted with an additional semi-solid pool modeled for Yb-HPDO3A by a super-lorentzian line shape. The Bloch-McConnell equation was solved using either measured or published input values (Supplementary Table 1)^36^,^37^. The exchange rates of the two agents were estimated using the Hanes-Woolf linear QUESP method^38^. The exchange rate of Eu-HPDO3A was estimated at ~5,000 s^−1^ at 18 ppm. No significant changes in spectra were evident with changes in exchange rates of <500 s^−1^. For Yb-HPDO3A two exchange sites were detected with ~10,000 s^−1^ at 69 ppm and 15,000 s^−1^ at 97 ppm.

For Eu-HPDO3A, the presaturation pulse was initially 1.5 s long, with a RF *B*_1_ intensity of 15 μT for solution phantoms. Based on the MT simulations, parameters were then adjusted for *in vivo* experiments to a presaturation pulse 800 ms long, with a RF *B*_1_ intensity of 23 μT. For Yb-HPDO3A, the presaturation pulse was initially 1.5 s, with a RF *B*_1_ intensity of 20 μT, and adjusted for *in vivo* experiments to 600 ms, with a RF *B*_1_ intensity of 56 μT.

#### Image processing

Eu-HPDO3A images (18 ppm) were corrected for B0 field inhomogeneity on a pixel-by-pixel basis by using the WASSR (Water Saturation Shift Referencing) method^39^. A B0 field map was generated for each CEST scan using a rapid acquisition relaxation enhancement (RARE factor of 32) spin echo sequence with TE of 3.8 ms and TR of 3 s. The presaturation pulse was applied at 0.1 μT for 1 ms at ±0.3 ppm offset with 0.04 ppm per step. In order to obtain an accurate water frequency for each voxel, a maximum symmetry algorithm was applied with spline-fitting being used to produce a z spectra for each voxel^40^. The resultant map was incorporated into the z spectra calculations to produce a voxel-by-voxel correction. No B0 field inhomogeneity was evident in Yb-HPDO3A z-spectra at 97 ppm, hence no correction for B0 inhomogeneity was applied. For both agents, the area-under-the-curve (AUC) method^41^ was used to generate CEST images for overlays, as this reduced spurious background noise compared to the more commonly used a line-by-line subtraction method (Supplementary Fig. 4). For Eu-HPDO3A, acquired data from offsets at ±16 ppm, ±17 ppm, ±18 ppm, ±19 ppm, ±20 ppm were used to calculate the AUC, whereas ±95 ppm, ±96 ppm, ±97 ppm, ±98 ppm and ±99 ppm were used for Yb-HPDO3A.

### *In vivo* cell implantation and imaging

All procedures were performed in accordance with protocols approved by the Institutional Animal Care and Use Committee (IACUC) of the University of Pittsburgh, as well as NIH guidelines.

#### Middle cerebral artery occlusion (MCAo)

Male Sprague-Dawley rats (180–200 g, Taconic) underwent MCAo surgery after one week’s acclimatization^42^. Under isoflurane anesthesia (4% for induction, 2% for maintenance), via the common carotid artery the right MCA was occluded for 70 min by insertion of a propylene filament (Doccol Corporation, USA). During the recovery period of two weeks, animals were monitored daily for their post-operative recovery and neurological deficits^42^.

#### MR imaging

To implant cells into the stroke cavity, animals underwent MR imaging 12 days post-MCAo. A T_2_-weighted MR image (FSEMS: TR = 6.1 s, TE = 70.78 ms, n = 8, 30 × 30 mm FOV, 192 × 192 matrix, 0.5 mm slice thickness) was acquired to exclude unlesioned animals, as well as to measure the volume and location of the stroke lesion^43^. T_2_-weighted images also served as anatomical reference for CEST image overlays. For CEST images, offsets for imaging were acquired on the specific offset resonance, as well as 2 images either side at 1 ppm for Eu-HPDO3A (±16 ppm, ±17 ppm, ±18 ppm, ±19 ppm, ±20 ppm) and Yb-HPDO3A (±95 ppm, ±96 ppm, ±97 ppm, ±98 ppm and ±99 ppm). Additional reference points for calculations were also acquired at 0 and −300 ppm. SAR effects were minimized by the use of a long TR to allow energy dissipation and temperature remained within FDA guidelines of maximum 1 °C whole body heating due to SAR effects^44^ (Supplementary Fig. 3B). The same acquisition strategy was also followed 1 day post-implantation of labeled or unlabeled NSCs/ECs.

#### Cell Implantation

The stroke cavity was identified on T_2_-weighted MR images as a hyperintensity. The volume of this was measured from thresholded images of 1 standard deviation above the corresponding undamaged hemisphere^45^. On the day of implantation, labeled and unlabeled cells were suspended at 160,000 Eu-HPDO3A STROC05 cells/μL and 40,000 Yb-HPDO3A D3 cells/μL in PBS. Animals were placed in a stereotactic frame (Kopf), and two burr holes (one for injection and one for fluid drainage) were made in the skull at the MRI-derived coordinates^46^. A sterile disposable 23 G needle was inserted slowly by hand into the drainage site, and a 250 μL Hamilton needle containing the cell suspension was inserted into the injection site. The material was then injected using a micro pump (Micro4, WPI) at 10 μL/min to a total volume equal to that of the cavity. After completion, the needles were retracted and bone wax used to fill drill holes. Animals were given analgesic and fluid replacement and the wound was sutured.

#### Perfusion

Immediately after imaging 1 day post-implantation, animals were sacrificed using Fatal Plus (Henry Schein) and underwent transcardial perfusion-fixation using 4% Paraformaldehyde (PFA, Electron Microscopy Sciences). Brains were then removed, and placed in 4% PFA overnight to ensure complete fixation.

### Immunohistochemistry

Brains were cryoprotected by placing into 30% sucrose for 3–4 days before being sectioned using a cryostat (Leica) at 50 μm thickness. Sections were then washed three times with PBS before being incubated in blocking solution (PBS + 0.5% Triton X100, Sigma +10% Normal Goat Serum, Vector Labs) for 1 hr at room temperature. Primary antibodies were then diluted in blocking solution and incubated with sections overnight at 4 °C. Antibodies used were Human Nuclear Antigen (1:400, HNA, Millipore,), Glial Fibrillary Acidic Protein (GFAP, 1:3000, Sigma,) and CD31 (1:250, Abcam). Sections were then washed 3x PBS before being incubated for 1 hr at room temperature with appropriate AlexaFluor secondary antibodies (1:500, Life Technologies). Sections were then stained with Hoechst (Sigma) as a nuclear counterstain and coverslipped using Vectashield mounting medium (Vector Labs). Images were acquired using an AxioImager M2 microscope (Zeiss) with StereoImager software (MBF). Whole hemisphere images were acquired using the Virtual Tissue software module (MBF). To capture the innate fluorescence of Eu-HPDO3A, a custom filter set was used (Chroma Technology, excitation filter 300–400 nm, bandpass emission filter 585–665 nm).

### Statistical analyses

Statistical analysis of cell counts and measurements were performed on mean values by using two-way analysis of variance (ANOVA) followed by Bonferroni post-hoc analysis and expressed as means ± standard error of means (SEM) with Prism 4 (GraphPad). A p value of <.05 was considered significant.

## Additional Information

**How to cite this article**: Nicholls, F. J. *et al.* Simultaneous MR imaging for tissue engineering in a rat model of stroke. *Sci. Rep.* **5**, 14597; doi: 10.1038/srep14597 (2015).

## Supplementary Material

Supplementary Information
