# Supplementary material for: Simultaneous MR imaging for tissue engineering in a rat model of stroke
Source: Sci Rep. 2015 Sep 30;5:14597. doi: 10.1038/srep14597 (PMC4588587; doi:10.1038/srep14597)
Supplement: Supplementary Information [file srep14597-s1.pdf]

Supplementary Material

Simultaneous MR imaging for tissue engineering in a rat model of stroke.

Francesca J. Nicholls[1,2,4,], Wen Ling[1], Giuseppe Ferrauto[5], Silvio Aime[5], Michel Modo[1,2,3]

[1]Department of Radiology, [2]McGowan Institute for Regenerative Medicine and [3]Department of Bioengineering, University of Pittsburgh, Pittsburgh, PA; [4]Institute of Psychiatry, Psychology and Neuroscience, King's College London, UK; [5]Molecular Imaging Center, Department of Chemistry, University of Torino, Italy

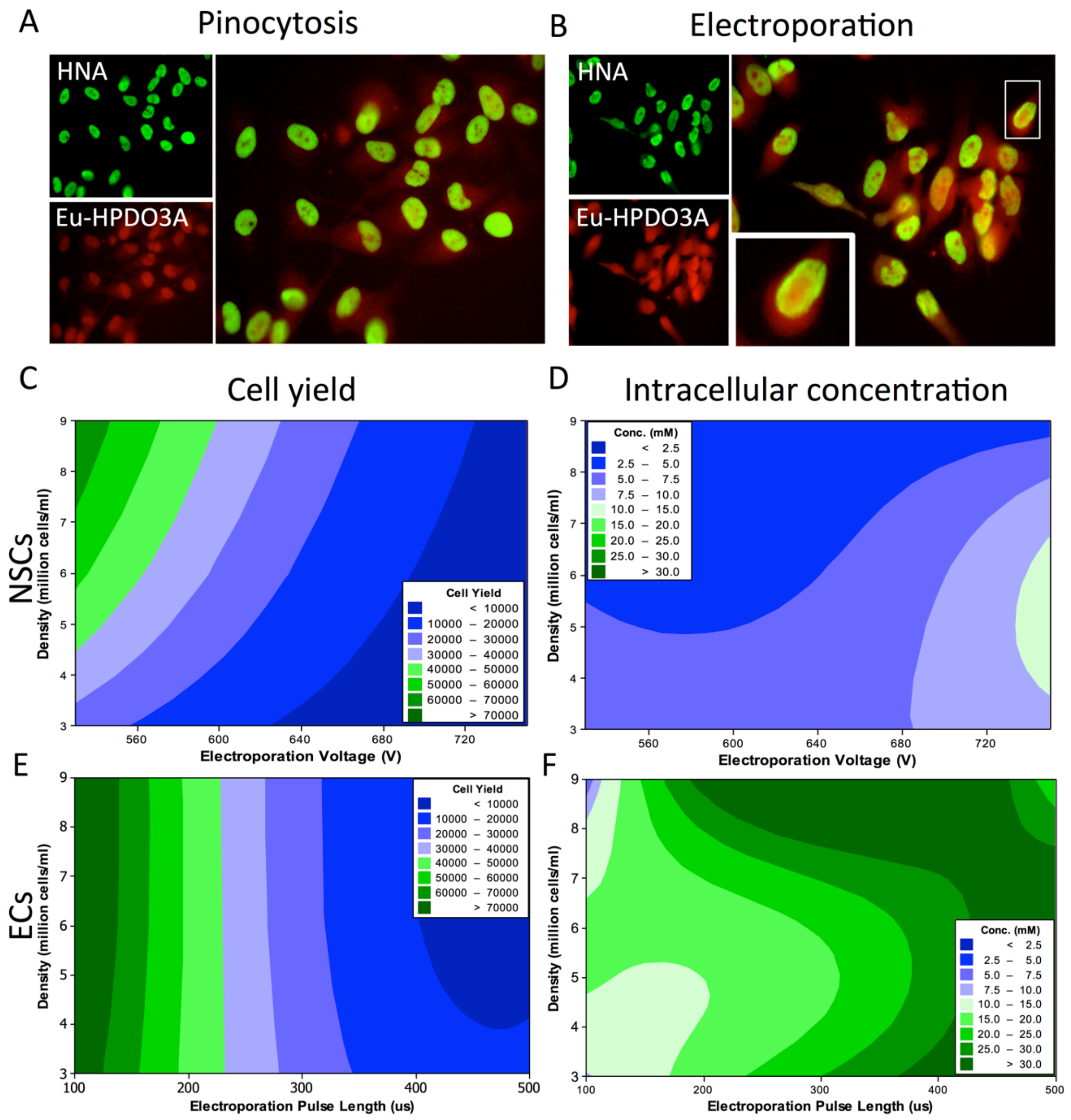

Supplementary Figure 1. Concept maps of cell uptake. Visualization of Eu-HPDO3A uptake through pinocytosis **(A)** and electroporation **(B)**. Concept maps of Eu-HPDO3A incorporation into NSCs indicate a synergistic effect of cell density and voltage affecting cell yield **(C)**, as well as intracellular concentration of agent **(D)**. Cell yield here reflect the number of surviving cells that could be harvested after 24 hours of re-plating. In contrast, for ECs, there was a clear linear relationship between pulse length used for electroporation and cell yield **(E)** with longer pulses being more detrimental to cells. In contrast to cell yield, cellular incorporation was influenced by both cell density and pulse length **(F)**. A potential impact on cell proliferation of the electroporation procedure was assessed by Ki67 staining. At 1 day post-electroporation there was little difference in Ki67 staining (in green) between electroporated and non-electroporated (control) NSCs **(G)**. A statistical

comparison further indicated no effect of electroporation on proliferation in NSCs, but at day 7 a significant 24% decrease ($p<.001$) was evident in ECs.

A

Figure	Voltage (V)			Pulse length (μs)			Cell density (x 10^6 cells/ml)			Agent (mM)		
B	100	530	1000	100			3			1		
C	530			15	100	300	3			1		
D	530			100			2	3	4	1		
E	530			100			3			0.5	1	5

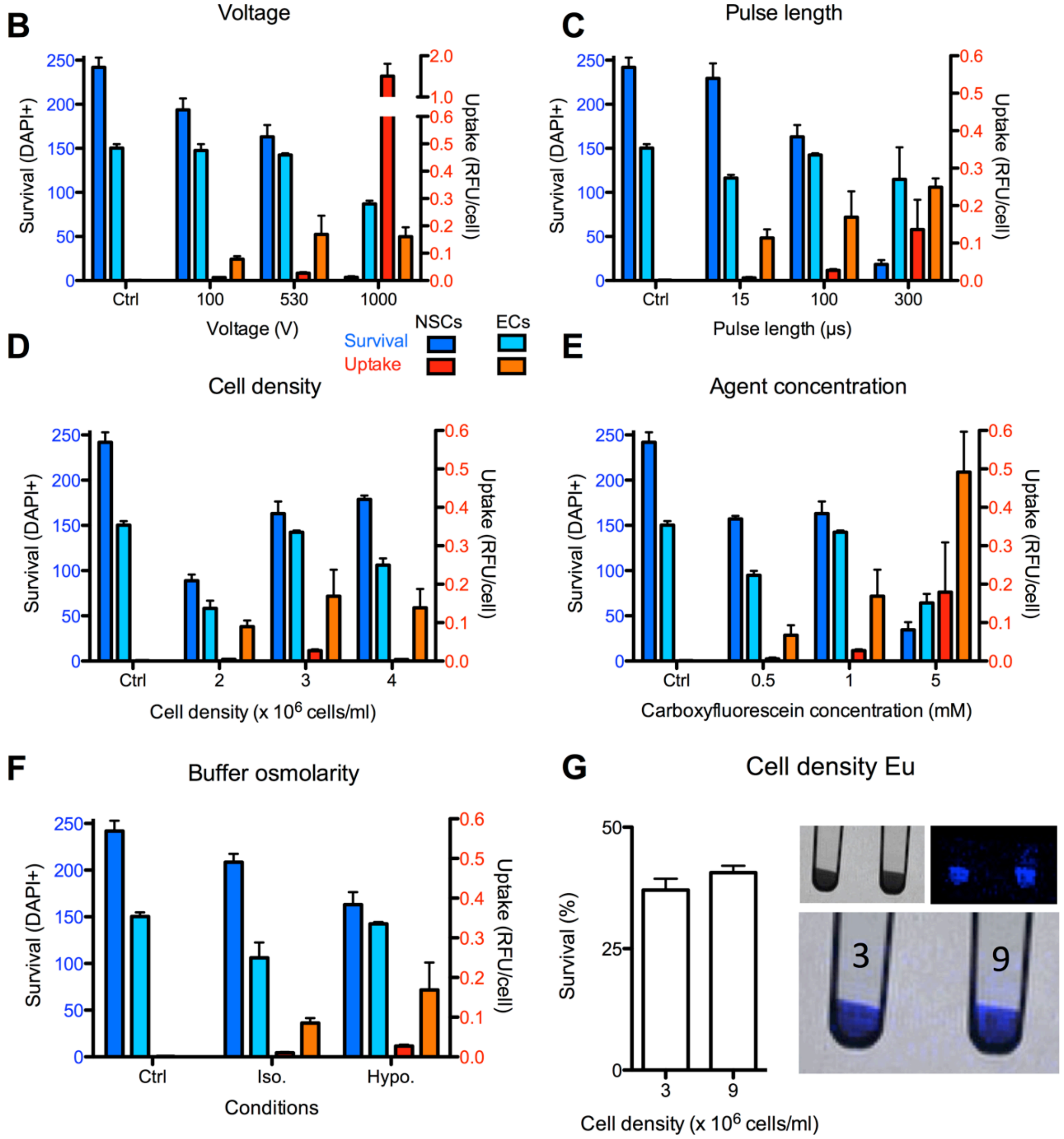

Supplementary Figure 2. Optimization of electroporation conditions. The efficiency of electroporation is determined by a variety of factors that can be varied to achieve an optimal uptake while maintain an acceptable cell yield. Values used for optimization are charted in (**A**). Varying the voltage of the electroporation pulse **(B)**

showed little effect of agent uptake in ECs (D3 cells, orange bars), but a dramatic increase in uptake for NSCs (STROC05, red bars), but also a decrease in survival. Increasing the length of the pulse **(C)** showed an increase in agent uptake into ECs, with no significant effect on cell survival, but for NSCs showed a small increase in agent uptake with a large drop in cell survival. Increasing the cell density during electroporation **(D)** appeared to have little effect on agent uptake, but increased cell survival for both cell types. Increasing agent concentration **(E)** showed an increase in agent uptake for both cell types, but also a decrease in cell survival. The use of hypo-osmolar buffer increased uptake in both NSCs and ECs compares to iso-osmolar buffer, whereas cell survival was slightly decreased for NSCs, and slightly increased for ECs **(F)**. The use of a higher cell density significantly improves the throughput of the electroporation procedure, so the use of a much higher cell density ($9x10^6$ cells/mL) was also assessed for Eu agent in NSCs, and had little effect on cell survival or CEST signal **(G)**.

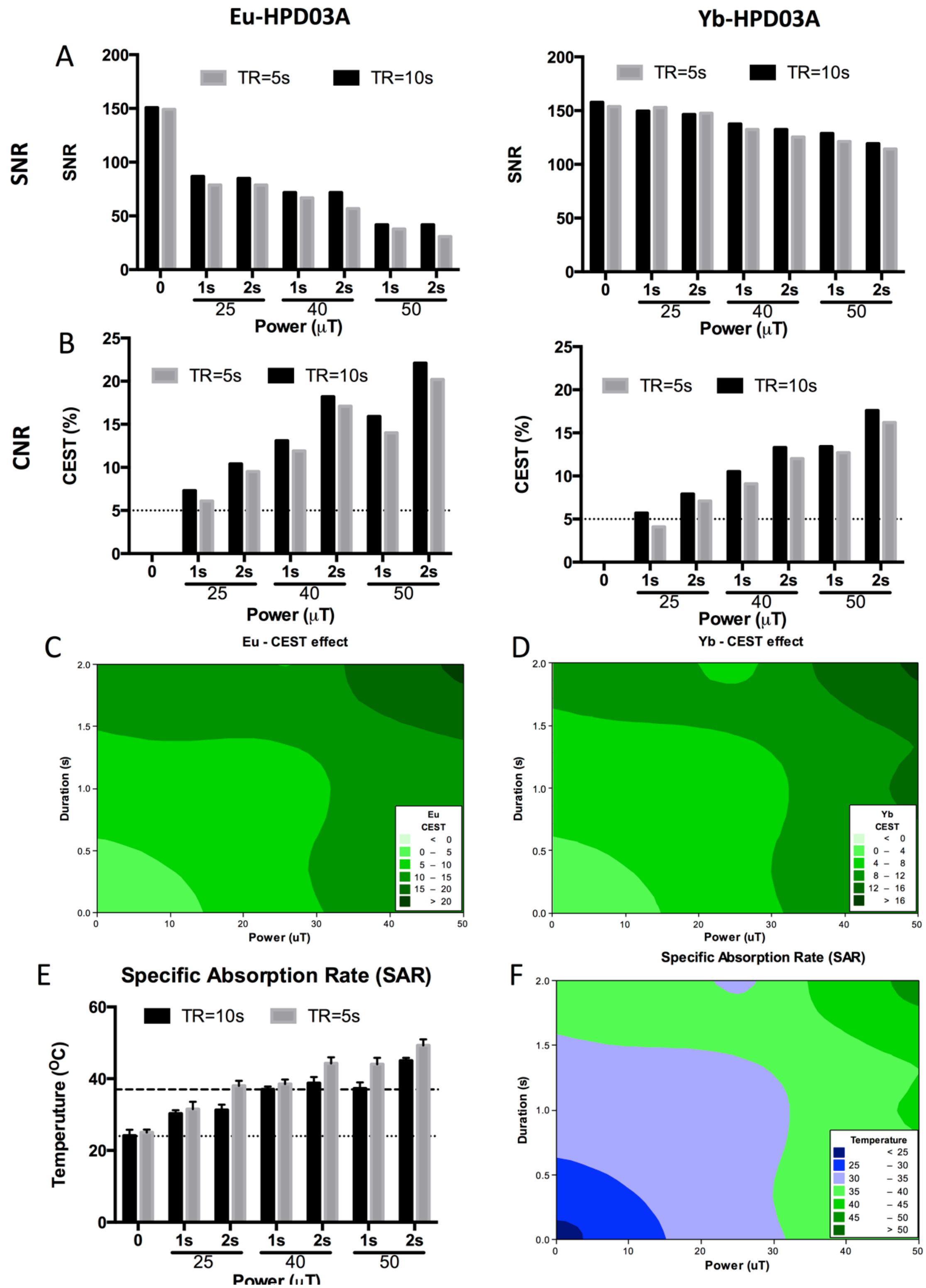

Supplementary Figure 3. Optimization of imaging parameters. To ensure the feasibility of paraCEST imaging, it is essential to balance the signal-to-noise ratio (SNR) and contrast-to-noise ratio (CNR) against potential deleterious heating effects due the specific absorption rate (SAR). Power, length of pulse and repetition time

(TR) were therefore arrayed to determine their effect on SNR, CNS and SAR. As expected, with increasing power SNR decreased **(A)** for both Eu-HPDO3A and Yb-HPD03A, but CNR increased **(B).** TR and pulse length had mostly negligible effects. Contour maps of the arrayed parameters indicated nevertheless an interaction between pulse length and power on the CEST effect for both Eu-HPDO3A **(C)** and Yb-HPDO3A **(D)**. By imposing physiologically acceptable conditions (temperature <38 °C), it was evident that acquisition parameters >40 μT with a 2 s pulse length would potentially be deleterious to in vivo studies **(E)**. A contour map again highlighted the non-linear nature of power and pulse length affecting heating of the sample **(F)**.

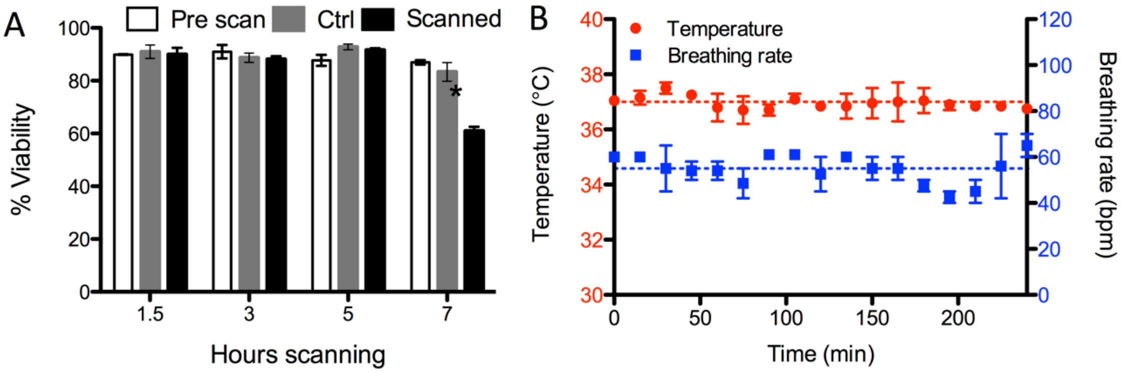

Supplementary Figure 4. Physiological considerations of paraCEST imaging. **A.** To ensure that imaging conditions do not adversely affect cells implanted with paraCEST agents, NSCs labeled with Eu-HPDO3A were placed in the scanner and imaged for different amounts of time (1.5, 3, 5 and 7 hrs) and compared to pre-scan viability and cells on the bench (Ctrl=controls). Only with 7 hrs of scanning, an effect on viability was evident. **B.** A further concern with paraCEST imaging are potential heating effects due to Specific Absorption Rate (SAR). To this end animals systemic temperature and breathing rate were monitored throughout scanning. No physiological effects were apparent using the current acquisition paradigm.

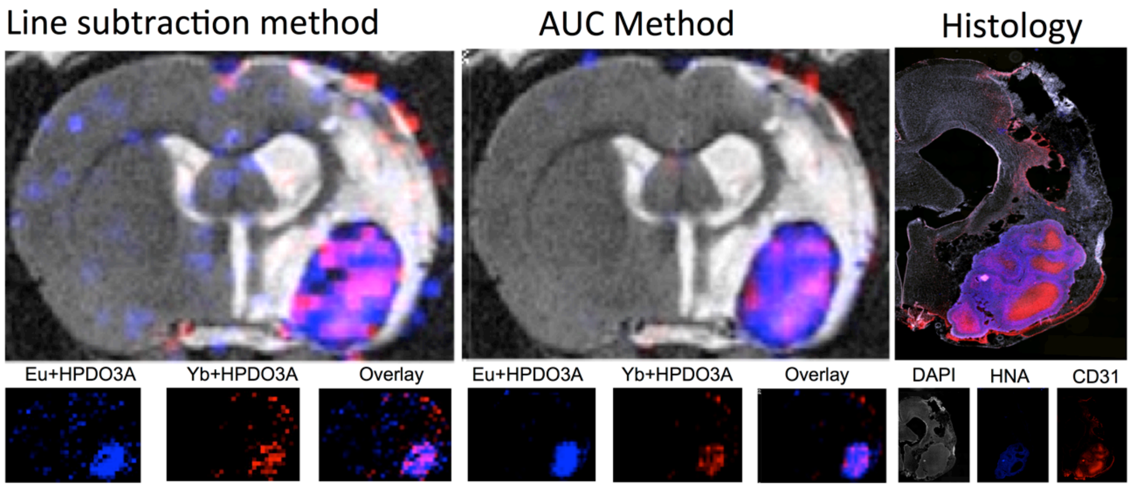

Supplementary Figure 5. Processing of paraCEST images. The conventional line-by-line subtraction method to calculate asymmetry for each point leads to a significant number of false positive voxels. In contrast, by calculating the area-under-the-curve (AUC) for all points on the negative and positive side afforded a significant reduction in the number of spurious voxels. However, it also resulted in an overall loss of effect size for voxels containing paraCEST agent.

	Free	Biol. Mol	Eu 18ppm	Yb 69ppm	Yb 97ppm
T1 (s)	1.4	0.1	0.77	0.77	0.77
T2 (s)	0.04	0.0000092	0.033	0.033	0.033
Δωi (offset, ppm)	0	0	18	69	97
Ratio	0.92	0.08	0.0001	0.0001	0.0001
k to free water (measured, s^{-1})	-	50	5,000	10,000	15,000
k from free water (calculated, s^{-1})	-	4.35	0.54	1.09	1.63
RF power (μT)	-	-	28	48	48
RF duration (s)	-	-	0.8	1	1

Supplementary Table 1. Parameters used for Bloch simulations of MT effects in brain tissue. Shaded areas report values for in vivo experiments.
